# Supplementary material for: Comprehensive Annotation and Functional Exploration of MicroRNAs in Lettuce
Source: Front Plant Sci. 2021 Dec 24;12:781836. doi: 10.3389/fpls.2021.781836 (PMC8739914; doi:10.3389/fpls.2021.781836)
Supplement: Supplementary file 3 [file Data_Sheet_9.ZIP › GSM2230753.plot/Lsa-miR168a_Lsat_1_v5_gn_8_261.1_1295_TPlot.pdf]

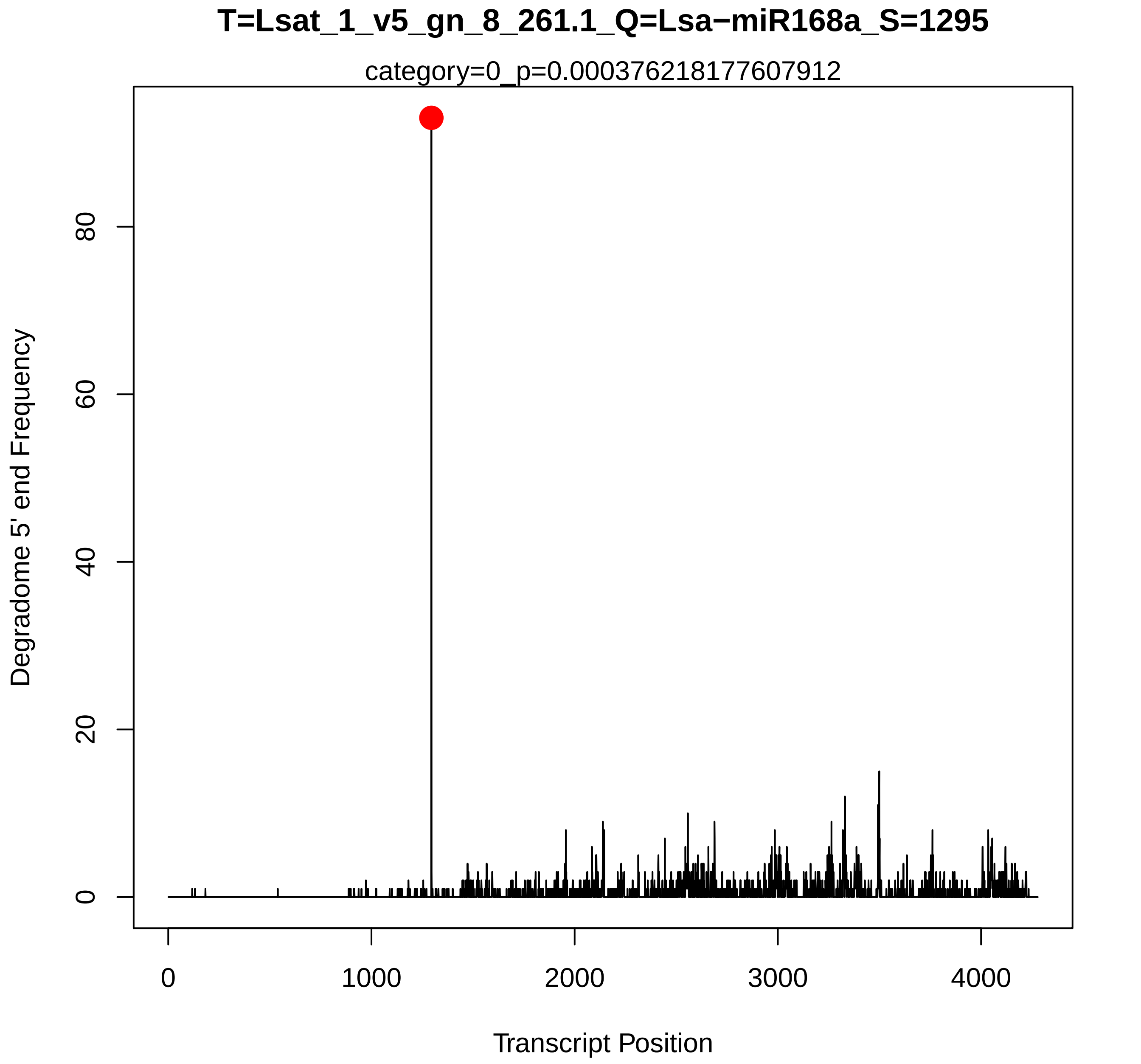
T=Lsat_1_v5_gn_8_261.1_Q=Lsa−miR168a_S=1295
category=0_p=0.000376218177607912
Degradome 5' end Frequency
0
20
40
60
80
0
1000
2000
3000
4000
Transcript Position
